# Supporting Midwifery Students During Clinical Practice: Results of a Systematic Scoping Review

**DOI:** 10.2196/36380

**Published:** 2023-04-21

**Authors:** Hafaza Amod, Sipho Wellington Mkhize

**Affiliations:** 1 School of Nursing and Public Health College of Health Sciences, University of KwaZulu-Natal Durban South Africa

**Keywords:** clinical support, mentorship training program, midwifery clinical education, midwife, midwifery, mentor, mentorship, clinical education training, clinical support, midwifery student, South Africa, Africa, framework, medical education

## Abstract

**Background:**

Midwifery educators are highly concerned about the quality of clinical support offered to midwifery students during clinical placement. The unpreparedness of midwifery practitioners in mentorship roles and responsibilities affects the competence levels of the next-generation midwives being produced.

**Objective:**

The aim of this paper is to highlight various clinical support interventions to support midwifery students globally and propose a framework to guide mentorship training in South Africa.

**Methods:**

This paper adopts a mixed methodology approach guided by the Arksey and O’Malley framework. Keywords such as midwifery students, clinical support, mentorship, preceptorship, and midwifery clinical practice were used during the literature search. The review included primary quantitative, qualitative, and mixed methods design papers published between 2010 and 2020, and studies on clinical support interventions available to midwifery students during clinical placement. The search strategy followed a 3-stage system of title, abstract, and full-text screening using inclusion and exclusion criteria. All included papers were quality appraised with a mixed methods appraisal tool. Extracted data were analyzed and presented in themes following a thematic content analysis approach.

**Results:**

The screening results attained 10 papers for data extraction. In total, 7 of the 10 (70%) studies implemented a mentorship training program, 2 (20%) used a training workshop, and 1 (10%) used an intervention guide to support midwifery students in clinical practice. Of these 10 papers, 5 were qualitative, 4 mixed methods, and 1 quantitative in approach. In total, 9 of the 10 (90%) studies were conducted in high-income countries with only 1 study done in Uganda but supported by the United Kingdom. The quality of included papers ranged between 50% and 100%, showing moderate to high appraisal results. Significant findings highlighted that the responsibility of mentorship is shared between key role players (midwifery practitioners, students, and educators) and thus a 3-fold approach to mentorship. Mentorship training and support are essential to strengthen the clinical support of midwifery students during placement. The main findings produced 2 main themes and 2 subthemes each. The main themes included strengthening partnerships and consultation; and providing mentor support through training. The 4 subthemes were: establishing stronger partnerships between nursing education institutions and clinical facilities; improving consultation between midwifery educators, practitioners, and students; the quality of clinical support depends on the training content; and the training duration and structure. Hence, the researchers proposed these subthemes in a framework to guide mentorship training.

**Conclusions:**

Mentorship training and support for midwifery practitioners will likely strengthen the quality of midwifery clinical support. A framework to guide mentorship training will encourage midwifery educators to develop and conduct mentorship training with ease. More studies using quantitative approaches in research and related to midwifery clinical support are required in African countries.

**International Registered Report Identifier (IRRID):**

RR2-10.2196/29707

## Introduction

Supporting students placed at various clinical facilities is an essential component of learning during clinical practice. In clinical education programs, such as midwifery, clinical placement is a perfect opportunity to achieve the skills necessary to become a safe and competent practitioner. The quality of midwifery students graduating is the responsibility of both midwifery practitioners and educators [1]. The midwifery module in the undergraduate nursing program is a hands-on module that expects midwifery students to spend most of their module time in clinical practice [2]. Therefore, midwifery educators rely on midwifery practitioners to clinically prepare students for role-taking, hoping that new graduates become competent, safe, and independent practitioners.

Recent challenges in the health care system and its effects on the quality of clinical support offered to midwifery students have become a significant concern for midwifery educators globally [3-5]. High student enrollment rates have subsequently increased the teaching workloads of midwifery educators [6]. Additionally, challenges related to developments in nursing programs and the unexpected disruptions experienced during the COVID-19 pandemic have increased midwifery educators’ academic and clinical responsibilities. The corresponding increase in the number of students placed at clinical sites has also become a challenge for midwifery practitioners. Uncertainties about mentoring roles, negative feelings about teaching, time constraints, and dire staff shortages and resources have negatively affected the clinical support of midwifery students [3,6,7].

However, global efforts using various clinical support models, such as mentorship, preceptorship, and clinical supervision, have shown positive outcomes on midwifery students’ clinical learning and support [8-11]. Mentorship is a highly recommended means to provide the support that students require [9,11,12], and mentorship training programs to support midwifery practitioners in mentoring roles have shown numerous benefits globally [11,13]. Mentorship in maternity units is a direct relationship between the mentor (midwifery practitioner) and the mentee (midwifery student). Midwifery practitioners who are either not trained or inadequately supported in mentorship roles experience difficulties in supervising students [12,14,15] and, as a result, feel unprepared to share the responsibility of mentoring students [2,16,17]. Lack of support for mentors in maternity departments is a global challenge [18]. Clear guidance on how to conduct mentorship training and a need to identify interventions to support midwifery practitioners in mentorship is likely to improve the clinical support of midwifery students in clinical practice. Disregarding mentorship improvements poses the risk of employing unprepared and unsafe practitioners who are detrimental to health care outcomes. This review aims to identify clinical support interventions for midwifery students globally and develop a framework to guide mentorship training in South Africa.

## Methods

### Study Design

This systematic scoping review followed a protocol developed to analyze the evidence on interventions to strengthen the clinical support of midwifery students during clinical placements [4]. The review followed a population, concept, and context framework [19]. The review focused on the concept of the clinical support available to midwifery students (population) in clinical placements in a global context.

### Identifying the Research Question

This review answers the research question what interventions are available to strengthen the current clinical support for midwifery students globally? By identifying and analyzing the clinical support interventions available on a global platform, the researchers desired to integrate these interventions to develop a new framework to guide mentorship training in South Africa.

### Search Strategy

The retrieval of records was through database searching conducted between September 2019 and March 2020. Hence, this review followed the PRISMA (Preferred Reporting Items for Systematic Reviews and Meta-Analyses) flowchart [20]. The search strategy included keywords midwifery students, clinical support, mentorship, and midwifery clinical practice. The search was refined to English and confined to the last 10 years (January 2010 to August 2020) to ensure only current and updated clinical support interventions for this review. The review included a hand search through the main published papers and citations from the “related literature” list.

Electronic databases used for this review included (1) EBSCOHost (CINAHL, MEDLINE, Health Source: Nursing/Academic Edition) using boolean terms such as midwifery and clinical support, midwifery and mentorship, midwifery and clinical supervision, and midwifery and preceptorship; (2) PubMed and Science Direct included MeSH (Medical Subject Headings) terms such as midwifery students and clinical support, or midwifery students and mentorship, or midwifery students and clinical supervision; and (3) Google and Google Scholar used keywords such as midwifery students in undergraduate nursing programs, midwifery students and clinical support, mentorship in midwifery, and midwifery clinical practice and clinical supervision.

The librarian assisted with retrieving full-text papers not found on the website. All researchers kept an electronic record of retrieved papers.

### Study Selection Process

The search strategy followed a 3-stage system of title screening, abstract screening, and full-text screening. The selection included qualitative, quantitative, and mixed methods papers published in peer-review journals. All selected papers were exported to an EndNote (Clarivate, 2020) library. Duplications were removed from the list. The primary investigator and an independent collaborator screened all saved abstracts using a standardized Google Form as a tool. Both the primary investigator and the independent collaborator applied the inclusion and exclusion criteria developed for this search.

Inclusion criteria include (1) only primary studies conducted between 2010 and 2020; (2) papers that used qualitative, quantitative, or mixed methods approaches; (3) papers that present programs, training, or interventions related to clinical support such as mentorship, preceptorship, and clinical supervision; and (4) papers available in the English language. Exclusion criteria include (1) studies that did not include a program, training, or intervention; (2) papers that were reviews; and (3) studies related to nurses in the general, community, and psychiatry nursing disciplines.

All papers selected from the abstract-screening stage were eligible for a full-text paper screening process using another standardized Google Form. Both the primary investigator and the research collaborator worked independently to screen all retrieved papers and compiled a report of both the abstract and full-text screening. A third reviewer (the research supervisor) was available to resolve any discrepancies; however, there were none at the time. The involvement of 3 reviewers prevented bias in the selection of papers. All selected papers from the screening process were saved in an EndNote software folder.

### Quality Appraisal

All included studies were quality appraised using a mixed methods appraisal tool [21]. The intention was to retrieve high-quality papers related to the topic, avoid reading flawed literature, and prevent bias or untrustworthy information, which is the essence of conducting a systematic scoping review.

### Data Charting and Analysis

This review identified papers, which included clinical support interventions. The data charting variables, included (1) the author’s name, (2) the year of publication, (3) the aims of the study, (4) intervention outcomes, and (5) the most significant findings.

A desktop review of included papers was followed by a thematic content analysis approach [21]. Data were organized into meaning units, coded, and presented as themes and subthemes.

### Ethics Approval

Ethical approval was obtained from the Human and Social Science Research Ethics Committee of the University of KwaZulu-Natal (HSS/1509/018M).

## Results

The results are presented as the screening results and the data extraction results.

### Screening Results

**Figure 1:**
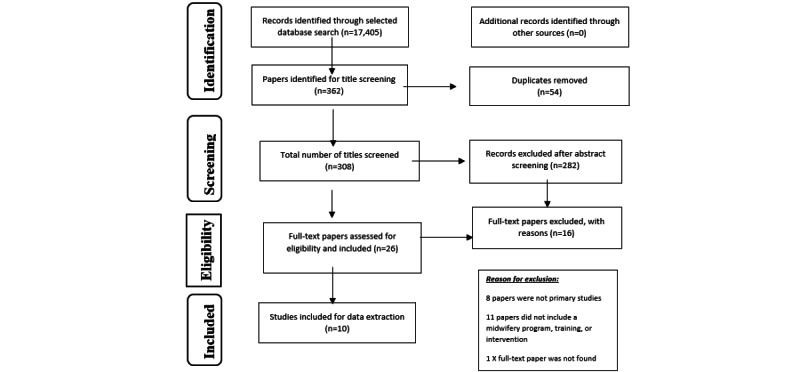
The screening results presented in a PRISMA (Preferred Reporting Items for Systematic Reviews and Meta-Analyses) flow diagram [20].

The researcher selected only papers from primary studies for this review and adopted the PRISMA flowchart [20]. The result of the screening process is shown in Figure 1. Screening results include the study characteristics (the research approaches and the study settings) and the quality of included papers.

### Characteristics and Quality of the Included Papers

#### Research Approaches

There were 5 qualitative papers [22-26], 4 mixed methods papers [11,13,27,28], and 1 quantitative paper [29].

#### Study Settings

From across the globe, 4 studies were in the United Kingdom [13,22,23,27], 1 in Scotland [26], 2 in Australia [24,28], 1 in the United States [25], 1 in New Zealand [29], and lastly, 1 study in Uganda partnered with the United Kingdom [11].

#### Quality Assessment

Ten papers remained for data extraction, and the quality assessment of these papers was according to the research approaches selected in these primary studies. Hence, the mixed methods appraisal tool was selected to audit these papers. In total, 5 of the 10 papers (50%) were qualitative, of which 3 scored 100% and 2 scored 75%, showing high-quality values. The quality of 4 mixed method designs showed scores between 50% and 100%, and the remaining quantitative design paper scored 100%. These results indicated that all 10 papers were of high quality and complemented the purpose of conducting a systematic scoping review.

### Data Extraction Results

Ten papers published between 2010 and 2020 remained for data extraction. The objective of this review was to identify interventions to support midwifery students during clinical practice. Data charting variables were applied to extract data during this stage. Table 1 shows the data extraction results.

**Table 1:** Data extraction.

Authors and year	Aims of the study	Intervention used	Significant findings
Broad et al [22], 2011	To support preregistration midwifery students during clinical placement	A transition model of preceptorship	The intervention facilitated midwifery students learning in practice through the guidance of a preceptor. Increased confidence and competence of newly qualified midwives. Contributed to staff retention, increase co-operation, and quality of care given. Increased investment in health care and education.
Barker et al [23], 2011	To train and support the role of mentors in assessing clinical competence of midwifery students	Mentor support by PEFs^a^	Support for mentors is critical to improve student facilitation and support in clinical practice. Protected time was necessary for SOMs^b^ to attend workshops. The intervention showed that better patient care outcomes increased collaboration between mentors, PEFs, and university and improved mentor assessment skills.
Durham et al [27], 2012	To develop skills in mentorship using a developmental program	A developmental program to support mentors	The program promoted high standards of mentoring knowledge and skills and improved understanding and accountability of the mentorship roles. A tripartite role benefitted the institution and the SOM.
Clements et al [24], 2012	To evaluate the core elements of a transition support program for newly qualified midwives from undergraduate and postgraduate nursing program	A transition support program for midwives	A structured support during this transitional phase is necessary to ensure quality and safe practice of midwives. Supernumerary time was highly valued but not always available. Midwives appreciated study days, which allowed them to share their clinical experiences and debrief. The program promoted peer midwife to midwife support.
Thunes and Sekse [25], 2015	To gain a better understanding of midwifery students’ first encounter in the maternity wards and what was essential to them in the learning environment	A planned clinical practice approach	Student-mentor relationships are crucial for students’ achievements and learning outcomes. Midwifery students need to feel valued and included in the team, learning was based on students’ expectations, understanding, and previous experience. Mutual engagement with mentors is necessary.
Dixon et al [29], 2015	To explore the retention of new graduates in midwifery practice following participation in the MFYP^c^ program	An MFYP program	The program provided mentor support to new midwifery graduate and increased their confidence in the first year of practice as a registered midwife.
Moran and Banks [26], 2016	To explore the experiences and the value of “SOMs”	SOMs and the value they hold to this role	Mentors valued their role and found it to be essential for the supervision of midwifery students during clinical practice. Students value mentors for continuity, feedback, and planning.
Hogan et al [28], 2017	To explore the benefits of a peer mentoring program for midwifery students	A peer mentoring program in midwifery clinical placement	Benefits to the mentee—reduced anxiety of first-year students, smoother transition to clinical practice, mentors were encouraging, understanding, reassuring, and positive. Benefits to the mentor—building communication skills, self-confidence, and increased employability.
Kemp et al [11], 2018	To develop a model of mentorship for Ugandan midwifery students to improve the quality of midwifery care	The MOMENTUM^d^ project 2015-2017	Showed improved knowledge, skills, and attitudes of students and mentors. Improved audit scores at clinical sites. Improved confidence; however, mentors did not assess students’ clinical skills in practice.
Tweedie et al [13], 2019	To evaluate the model of coaching and collaborative learning and the role of the clinical education midwife	Collaborative coaching and learning model adapted from the CLiP^e^ model by Lobo et al [30], 2014	Improved students’ confidence in knowledge and clinical and communication skills. Student support through a clinical education midwife. Ensured partnership between HEI^f^ and hospitals.

^a^PEF: practice education facilitator.

^b^SOM: sign-off mentor.

^c^MFYP: Midwifery First Year of Practice.

^d^MOMENTUM: Developing a Model of Mentorship for Ugandan Midwifery.

^e^CLiP: Collaborative Learning in Practice.

^f^HEI: higher education institution.

### Answering the Research Question

The objective of this review was to identify interventions available to strengthen the current clinical support for midwifery students globally. Interventions identified in this review included training programs, workshops, and one intervention guideline.

### Synthesis of Screening Results

#### Overview

In total, 7 of the 10 studies (70%) implemented mentorship or preceptorship programs [11,13,22,24,26,28,29]. Two studies (20%) conducted a training workshop [23,27], while only 1 study (10%) included an intervention guideline [25]. These interventions supported either midwifery students or clinical mentors during clinical placements. The benefits of using clinical support interventions showed improvements in students’ confidence levels, competence, and readiness for role-taking; it also revealed benefits for the clinical mentor in terms of improved mentorship knowledge, skills, and accountability [11,28]. Beyond these benefits, clinical support interventions show improved patient care outcomes [22,23] and collaborations between clinical facilities and nursing education institutions (NEIs) [13,23].

Meta-analysis of the significant findings was conducted to identify how interventions can be combined, adapted, and integrated to produce a more robust conclusion on strengthening midwifery students’ clinical support during practice. Six codes emanated from the significant findings, as seen in Textbox 1. The third reviewer verified the findings and the constructed codes. These codes included academic-service partnerships, collaboration and consultation, clinical support methods, clinical support guidelines, clinical support materials, and course content.

These constructed codes were further analyzed to identify a more intense understanding of how to strengthen mentorship in midwifery. The review adopted a thematic content analysis approach [21]. Overall, 2 themes, with 2 subthemes each, emerged from the analysis. These themes are essential to guide mentorship program development and sustainability.

**Coding of significant findings.**

**Academic-service partnership**

- Partnership between the clinical placement facility and the higher education institution is essential when designing an intervention for clinical support of midwifery students
- Partnership includes liaison between various stakeholders such as university educators, nurse managers, government personnel (if necessary), Mentors or preceptors or clinical facilitators, and expert advisory groups

**Collaboration and consultation**

- Continuous collaboration between the university and the hospital through a link lecturer is important
- Consultation with clinical mentors, midwifery students, students’ support services, quality assurance teams, and previous cohort of students

**Clinical support methods**

- Presentations ranging from 3 hours to half-day workshops for clinical mentors included case scenarios, Objective Structured Clinical Examinations for evaluating mentor knowledge and skills
- Structured clinical support program for students, which includes student rotation plans, supernumerary time, and study days. Includes support for clinical mentors from universities, colleges, colleagues, and senior managers
- Structured Midwifery First Year of Practice program for newly qualified midwives
- 10-day study program validated by the Nursing and Midwifery Council (NMC) guidelines
- Peer-to-peer mentoring—3-hour training of third-year students (clinical mentor)
- Pilot sampling of intervention was adopted in 2 studies

**Clinical support guidelines**

- NMC Standards framework for nursing and midwifery education (2018) [31]
- Australian and New Zealand Support Services Association Incorporated guidelines

**Materials used in clinical support training sessions**

- Workbooks, portfolios, booklets, information pack, and a toolkit

**Course content**

- Role of the clinical mentor and mentee—named preceptors
- Outline of the program—practical component or areas of practice or placement schedules or clinical rotations, study days or skills education days, relationship building, communication skills, feedback, and debriefing opportunities
- Professional issues—NMC guidelines or standards for mentors
- Responsibilities or role expectations of clinical mentors—include boundary restrictions
- Self-care or support services available and referrals

#### Theme 1: Strengthening Partnerships and Consultation

##### Overview

The included papers revealed that improved partnerships and consultations were vital in supporting students during clinical placement. This theme developed from 2 subthemes: establishing stronger partnerships between NEIs and clinical facilities and improving consultation between midwifery educators, practitioners, and students.

##### Subtheme 1.1: Establishing Stronger Partnerships Between Nursing Education Institutions and Clinical Facilities

In 2011, the transition model of preceptorship began through regular meetings between the nurse managers and heads of departments at NEIs [22]. This strategy aimed to link the education and practice setting through a preceptorship model, which assisted midwifery students in achieving the required clinical practice standards. This highlighted that collaboration between the health facility, the facilitator, and the NEI is the cornerstone for success in mentorship [13], especially when negotiating protected time for mentors to attend workshops [23] or conduct mentor skills training [27]. Support from liaison facilitators employed at hospital facilities and educators of higher education facilities helped mentors to gain confidence in teaching and supervising students in practice. Hence, strengthening partnerships between NEIs and clinical facilities will facilitate continued collaborations and thus improve the clinical support of midwifery students. The idea was well-supported in other studies included in this review [11,26,28].

##### Subtheme 1.2: Improving Consultation Between Midwifery Educators, Practitioners, and Students

The review revealed that main stakeholders such as the nurse managers, regional or placement coordinators, clinical preceptors or mentors, midwifery practitioners, practice educators, or link lecturers have their roles in supporting midwifery students in clinical placements. Six papers showed that knowing the role of the mentor or preceptor, a named preceptor, contact details, clinical rotation, study days, and supernumerary time were factors that influenced the degree of clinical support offered to students by midwifery practitioners [22-24,26-28]. In addition, the continuity in students’ support by the same preceptor with a planned or structured clinical plan influenced students’ learning outcomes [13,25]. These authors further recommended that mutual engagement, shared knowledge, and shared goals are imperative to improving students’ learning outcomes. Continuous relations between midwifery educators and practitioners should be encouraged because both share the responsibility of mentoring midwifery students during clinical practice.

Mentor relationships affect the students’ perceptions of clinical practice. Students felt they depended on mentors to teach, show, and help them [25], and mentors, too, became optimistic. They showed interest in students’ expectations and engaged with students through good teamwork and communication [26]. Furthermore, the mentor roles were valued because they played an essential role. Therefore, describing the mentors’ role and expectations is critical in the training program, and this should be clear at the training program’s onset [27].

#### Theme 2: Providing Mentor Support Through Training

##### Overview

Midwifery practitioners in clinical placements often feel unprepared to teach students due to the lack of training and support that is available to them. Without the necessary support and training, midwifery practitioners cannot fulfill a mentor’s expected roles and responsibilities. Hence, mentor support and training are vital ingredients to improve the clinical support of midwifery students during placement. Two subthemes, namely, the quality of clinical support depends on the training content; and the training duration and structure.

##### Subtheme 2.1: The Quality of Clinical Support Depends on the Training Content

Durham et al's [27] study showed that a developmental training program to support mentors in their role focused on the content of the course and included a theory and practical component to support this training. The training content may include discussions on roles and responsibilities, professional issues, and boundaries to mentorship [22]. Therefore, mentorship training programs should include the policies and guidelines that govern midwifery education, practice, and training. In this review, 9 of the 10 studies (90%) were in first-world countries and guided by the Nursing and Midwifery Council (NMC). One peer mentoring study used the “Australian and New Zealand Support Services Association Incorporated guidelines” for peer mentoring. The training included the program’s aims and objectives, the available resources, and a program evaluation [28]. According to Thunes and Sekse [25], mentorship training programs should have a planned clinical practice approach that emphasizes students’ knowledge, skills, and learning needs to provide an overview of the mentors’ expectations. Therefore, training courses for mentors should include information regarding student expectations of the midwifery curriculum, clinical practice requirements, and competencies to be achieved during clinical practice. Midwifery practitioners should be familiar with student requirements outlined in midwifery clinical workbooks and portfolios [27] or clinical booklets [28] to assist students to meet these requirements timeously. The content of training programs becomes critical to the success of mentorship. The information offered should ensure that training attendees become knowledgeable and skilled in their expected roles and responsibilities.

##### Subtheme 2.2: The Training Duration and Structure

This review identified clinical support interventions that range from a 3-hour face-to-face training session to a 10-day study program and extended to a 12-month program. Training sessions were either informal or unplanned or formal and planned and took place in the clinical placement site. Findings showed that mentors involved in informal, shorter, or fragmented training sessions could not attend all the sessions as they experienced challenges with leaving the wards and received poor support from senior colleagues and managers [23,24]. A well-planned and structured mentorship training program contributed to better clinical support outcomes [25,28]. Hence, the timing of mentorship training programs is vital to consider in line with ensuring that the program is well-planned, formalized, and nonfragmented.

### Developing a Framework

**Figure 2:**
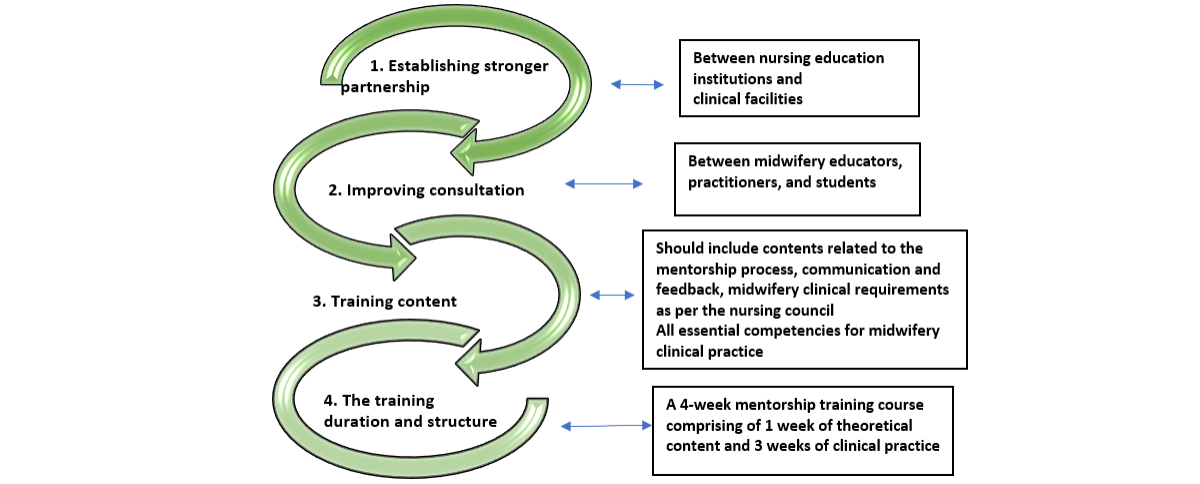
A framework to guide mentorship.

Themes identified in this systematic scoping review are the significant results emanating from tried and tested interventions of previous studies. Therefore, the results that were recurrently seen in the included studies guided the researchers to identify core considerations when planning and developing mentorship training programs. These 4 subthemes are foundational for supporting any mentorship training program, and hence, the researchers propose these subthemes as a framework (see Figure 2) to guide mentorship training.

## Discussion

### Principal Results

This review identified interventions to support midwifery students during clinical practice. Included interventions were mentor support programs, mentorship models, models of preceptorship, mentor developmental programs, and collaborative learning in practice models. Findings showed that mentorship was the most practiced intervention in supporting students globally, producing benefits to both students and mentors. Additionally, the benefits of mentorship extended to improved patient care outcomes and collaborations between NEI and clinical facilities. However, mentorship training and support for midwifery practitioners who undertake the mentor role are not well established, and concerns over graduates’ competence are worrisome. Therefore, it is necessary to follow clear guidance in developing successful mentorship training programs. The analysis of included papers highlighted essential aspects to consider when developing mentorship training programs. This involves strengthening partnership and consultation by establishing more robust relationships between NEI and clinical facilities and subsequently improving consultations between midwifery educators, practitioners, and students. Providing mentor support through training is essential, and therefore, the training content, structure, and duration of the mentorship training should accommodate clinical expectations.

### Comparison of Prior Work

The quality of clinical support for midwifery students is a concern despite efforts toward improvements. This review showed that mentorship is the blueprint for supporting midwifery students to achieve the expected competence needed to become safe and independent practitioners. Mentorship benefits are seen globally, especially in many developed countries, and are effective in clinically preparing students for role-taking [12]. Similarly, this review presented that the benefits of mentorship extend from midwifery students to practitioners, academics, and patients or clients. Therefore, nurse managers and heads of NEIs should support midwifery practitioners and educators, respectively, in this shared mentorship responsibility. Hence, partnerships and collaborations between NEIs and clinical placements are necessary.

Continued consultation opportunities contribute to a better understanding of students’ clinical expectations [22,23]. In 2011, trained sign-off mentors assisted midwifery students in achieving the requirements for clinical practice. However, these mentors experienced numerous challenges and felt inadequately prepared and supported in the role [23]. Subsequently, practice education facilitators were employed to support sign-off mentors in their roles [23].

In South Africa, midwifery educators and practitioners share the responsibilities of mentoring midwifery students during clinical placement. Improving consultation between midwifery educators (from NEIs) and midwifery practitioners (from clinical facilities) is needed to improve students’ support. Student-centered learning approach in higher education institutions promotes student responsibility and accountability for own learning outcomes. As a result, midwifery students understand that establishing good mentorship relationships with midwifery educators and practitioners is crucial in achieving clinical learning outcomes. In an attempt to review the current midwifery preregistration programs, the NMC supports and empowers students to become active or self-directed learners [32] as does the South African Nursing Council (SANC) [2,32].

The findings from this review highlighted the importance of conducting a well-structured mentorship training program. These programs should align with the learning objectives stipulated by nursing councils and NEIs. Hence, maintaining strong partnerships and regular consultation between relevant stakeholders (NEIs and clinical facilities) is necessary to improve the clinical support of midwifery students. Furthermore, the training program’s content should contain the students’ learning objectives, the process of mentorship, essential midwifery competencies, assessment and support materials, contact details of midwifery educators, and guidelines to follow during the mentorship process. Through content-specific and contextualized mentorship training programs and support, midwifery practitioners should be able to carry out mentorship roles and responsibilities with ease.

Empowering midwifery practitioners through mentorship training and support is advantageous to the quality of service provided at a clinical facility. Yet, clinical challenges remain a barrier to attend training workshops conducted off-site. Besides, too lengthy training programs are also an inconvenience in fragmented working schedules. Therefore, on-site, short-term, on-the-job mentorship training approaches that integrate theory-related instruction are likely to complement a “hands-on” approach in clinical mentorship.

### Strengths

Conducting systematic scoping reviews is a major strength in research as it ensures that only high-quality papers are included for data extraction. The review applied a mixed methodology, which provided a more detailed analysis of the findings. This review aims to identify the various interventions to strengthen midwifery clinical support and proposes a framework to guide mentorship training. The framework to guide mentorship training (Figure 2) is an investment to midwifery education and practice.

### Limitations

The limitations of the study are as follows. First, this review was restricted to clinical support interventions available to midwifery students only and may have limited the clinical support interventions available across nursing disciplines. However, the selected population of this review was midwifery students only and hence did not affect the study results. Second, a restricted timeframe over the last decade (2010 to 2020) may have excluded older but more applicable models of interventions. In view of this limitation, the results may have been short-played. Third, the review excluded the implications of mentorship to other categories of nurses, and hence, this should be explored further in future studies.

### Future Directions

The results of this review are likely to assist program developers and midwifery educators to participate in mentorship training and support programs. Strengthening mentorship through training opportunities for midwifery practitioners creates a platform to network and collaborates for the betterment of midwifery clinical practice and education. Given the limited papers retrieved from African countries in this review, there is a need for more research studies and publications on midwifery clinical education in African countries.

### Conclusions

Across the globe, mentorship training programs were the most common clinical support available to midwifery students. Mentorship in maternity departments is crucial, and mentors require the support of their colleagues, senior managers, and midwifery educators to ensure mentorship success. The ultimate success of mentorship lies in improved patient care outcomes. Therefore, mentorship training and support for midwifery students should not be side-lined because the safety of our patients is in the hands of these students currently in training.

Mentorship training and support programs alone are insufficient to meet role players’ needs. It is important to strengthen partnerships between NEIs and clinical facilities as it allows midwifery educators to become involved in the training and support of midwifery practitioners ceasing consultation and collaboration opportunities. By expanding and promoting engagements between midwifery students, practitioners, and educators, mentorship in midwifery becomes an equally important 3-fold shared responsibility, and this is the goal mentorship program developers want to achieve.

So, mentorship program developers want to advance the scope of mentorship. Attempts to revive mentorship training opportunities are necessary. Despite global attempts to strengthen mentorship, the competence of midwifery students produced remains a significant concern. The framework to guide mentorship training proposed in this review is likely to encourage midwifery educators to pursue more mentorship training opportunities with ease and hence, improve the quality of midwifery clinical education.

A structured mentorship training program to support midwifery practitioners in their mentorship roles and responsibilities is necessary to make improvements in the quality of clinical support. Midwifery students who are well-supported during clinical placement assures that the next generation of midwives are safe and competent practitioners who are likely to contribute to positive maternal health outcomes globally and in South Africa.

## Data Availability

All data generated and analyzed from this study will be available on request.

## Abbreviations

MeSH: Medical Subject Headings
NEI: nursing education institution
NMC: Nursing and Midwifery Council
PRISMA: Preferred Reporting Items for Systematic Reviews and Meta-Analyses
SANC: South African Nursing Council
